# Understanding the Factors Shaping the Effectiveness of Chinese Medical Team Programmes in Ghana: A Qualitative Study Using Bardosh’s Framework of Global Health Delivery

**DOI:** 10.34172/ijhpm.9320

**Published:** 2026-05-18

**Authors:** Emmanuel Kwasi Afriyie, Samuel Egyakwa Ankomah, Munawar Harun Koray, Sara Sulieman, Huijuan Liang, Dadong Wu, Dong (Roman) Xu

**Affiliations:** ^1^School of Public Health, Southern Medical University, Guangzhou, China.; ^2^Acacia Lab for Implementation Science, School of Health Management, Southern Medical University, Guangzhou, China.; ^3^Komfo Anokye Teaching Hospital, Kumasi, Ghana.; ^4^Department of Management, University of Cape Coast, Cape Coast, Ghana.; ^5^Upper West Region Health Directorate, Wa, Ghana.; ^6^Department of Pharmacy Practice, Faculty of Pharmacy, University of Khartoum, Khartoum, Sudan.; ^7^School of Healthcare Management, Inner Mongolia Medical University, Hohhot, China.; ^8^Shenzhen Maternity and Child Healthcare Hospital, Southern Medical University, Shenzhen, China.; ^9^SMU Institute for Global Health (SIGHT) and Center for World Health Organization Studies, School of Health Management and Dermatology Hospital of Southern Medical University (SMU), Guangzhou, China.

**Keywords:** Chinese Medical Teams, Ghana, Medical Aid, Global Health Delivery, Health Partnership, Qualitative Research

## Abstract

**Background:**

Chinese medical teams (CMTs) have long been a component of global health engagement in Africa, yet their effectiveness within national health systems remains under-examined.

**Methods:**

Drawing on Bardosh’s socio-anthropological framework of global health delivery, this qualitative study examines how CMTs function within Ghana’s public healthcare sector. Participants were purposively selected from key institutions in Ghana, including the Ministry of Health (MoH), two tertiary hospitals, and one district hospital hosting CMTs. Eighteen semi-structured interviews were conducted with Ghanaian clinicians, hospital administrators, national policy-makers, and Chinese medical staff. Policy-relevant documents from 2009 to 2024 were reviewed to supplement and triangulate the findings.

**Results:**

Based on Bardosh’s five domains, the analysis identifies several factors influencing programme effectiveness: the placement and facility-level integration of CMTs (terrain of intervention); cultural and linguistic disconnects affecting patient engagement (community agency); short-term rotations and fragmented collaboration with local staff (field staff strategies); limited adaptability and utilisation of donated medical technologies (socio-materiality); and weak institutional coordination and policy alignment (governance). Two additional themes—patient-centred care and continuity of healthcare delivery—emerged as important dimensions shaping the perceived value and limitations of the CMT model. The findings suggest that effectiveness is shaped not only by operational or resource-related factors but also by the extent to which foreign medical interventions are socially embedded, locally responsive, and institutionally aligned.

**Conclusion:**

These findings provide insights for the design of international health partnerships seeking to strengthen public health systems in low-resource settings through more participatory, coordinated, and locally embedded models of delivery.

## Background

**Key Messages**

**Implications for policy makers**

- The effectiveness of Chinese medical team (CMT) programmes is closely linked to how well they are integrated within host-country health systems, highlighting the importance of aligning CMT deployment with national health priorities and existing governance structures.
- The concentration of CMT activities in tertiary urban hospitals improves access to specialised care but may unintentionally reinforce existing urban–rural disparities in health service provision.
- Long-term sustainability of international medical assistance depends on institutional integration, including local capacity building, maintenance of medical technologies, and continuity of supportive infrastructure.
- International health partnerships benefit from moving beyond short-term or transactional aid towards co-constructed collaborations that prioritise mutual learning, accountability, and adaptive institutional relationships.

**Implications for the public**

In countries receiving international medical assistance, such as Ghana, foreign medical teams can provide specialised services, clinical training, and additional healthcare resources that may improve access to advanced treatment. However, the impact of such programmes is shaped not only by the availability of medical expertise but also by how well these teams are integrated into the local health system and communities. Factors such as language, cultural understanding, and collaboration with local healthcare professionals influence how patients experience these services. By highlighting these dynamics, this study helps readers better understand both the opportunities and limitations of international medical assistance programmes and the importance of sustainable partnerships that strengthen local health systems and benefit communities over the long term.

Since the 1960s, China’s medical aid has served as a strategic pillar of its foreign policy, evolving to address the changing health needs of low- and middle-income countries (LMICs).^1-5^ Much like historical health cooperation efforts by actors like the United States, United Kingdom, Germany, and Cuba in Africa,^6^ China has prioritised the deployment of Chinese medical teams (CMTs) across the continent, particularly since the early 2000s.^7-10^ These efforts have been institutionalised through the Forum on China-Africa Cooperation, which formally positioned medical diplomacy as a cornerstone of China’s soft power strategy, fostering mutual trust and development through shared health goals.^11,12^

 CMT programmes have encompassed infrastructure development, control of endemic diseases and emergency response. For instance, CMTs played a pivotal role in containing the 2014–2015 Ebola virus disease outbreak in Sierra Leone and Liberia.^13-15^ Additionally, the China-Tanzania Malaria Control Project (2015–2021), a two-phase initiative that integrated a locally-adapted malaria control strategy into Tanzania’s health system, demonstrated the feasibility of adapting China’s malaria-elimination model for high-burden settings.^16^ Furthermore, China pledged one billion COVID-19 vaccine doses to African nations during the 2021 Forum on China-Africa Cooperation summit.^17^ These actions collectively support the achievement of Sustainable Development Goals 3, 10, and 17^18^; and reflect China’s positioning as a provider of global public goods.^2,7,19^ However, while these efforts are framed as cooperative and developmental, scholars have also noted the political, symbolic and strategic interests embedded in China’s global health engagements, prompting calls for more empirical evaluations of their local impact.^1,5,20^

 Ghana, a West African nation with an estimated population of 34.6 million,^21^ has been a longstanding recipient of China’s medical aid since 2009.^7^ Despite its status as a regional economic hub, Ghana’s health system continues to face structural challenges, including underdeveloped infrastructure, health workforce shortages and persistent resource constraints.^22-25^ CMTs have been deployed to Ghana under bilateral agreements, primarily based in tertiary and district hospitals. Their work spans traditional Chinese medicine, training programmes, and surgical specialties such as cardiology under the “Heart to Heart” programme.^7,9,26^ Research indicates that these contributions have positively influenced clinical outcomes and health service delivery in Ghana. Nonetheless, challenges such as language barriers, cultural disconnects and logistical coordination issues continue to constrain the effectiveness of such programmes.^3,10,27^

 Despite the sustained presence of CMTs in Ghana, there remains limited empirical research on the systemic and contextual factors shaping their effectiveness within the public health sector. This qualitative study seeks to address that gap by examining how CMTs function within Ghana’s health system, guided by Bardosh’s socio-anthropological framework of global health delivery, which was developed to analyse factors influencing the effectiveness of global health interventions. In this study, “effectiveness” is understood in a qualitative and contextual sense, referring to the extent to which CMT programmes operate within and adapt to the local health system, including their ability to deliver services, build trust, and integrate with existing healthcare structures.

## Methods

###  Study Design

 An exploratory qualitative design was adopted to capture in-depth insights into the organisation, implementation and perceived effectiveness of CMT programmes within Ghana’s health system. Data were triangulated through semi-structured interviews with key informants and a review of relevant policy and institutional documents. The design and reporting of the study adhered to the Standards for Reporting Qualitative Research (SRQR)^28^ (Supplementary file 1, Table S1).

###  Guiding Framework

 Bardosh’s framework was adapted to guide the research process (See Figure). Originally developed through rapid ethnographic studies in Tanzania, Uganda and Zambia, the framework was designed to analyse large-scale neglected tropical disease interventions and offers a structured and robust tool for examining global health delivery across diverse settings.^29,30^ It comprises five interrelated domains: (1) the terrain of intervention, including geographical, environmental and seasonal conditions; (2) social difference and community agency, such as local knowledge, leadership structures and social cultural behaviours; (3) the strategies and incentives of field staff, addressing staff skills, motivation, and institutional support; (4) the socio-materiality of technology, referring to the properties, appropriateness and uptake of medical tools; and (5) the governance of interventions, encompassing bureaucracy, policy implementation and broader political dynamics.

**Figure:**
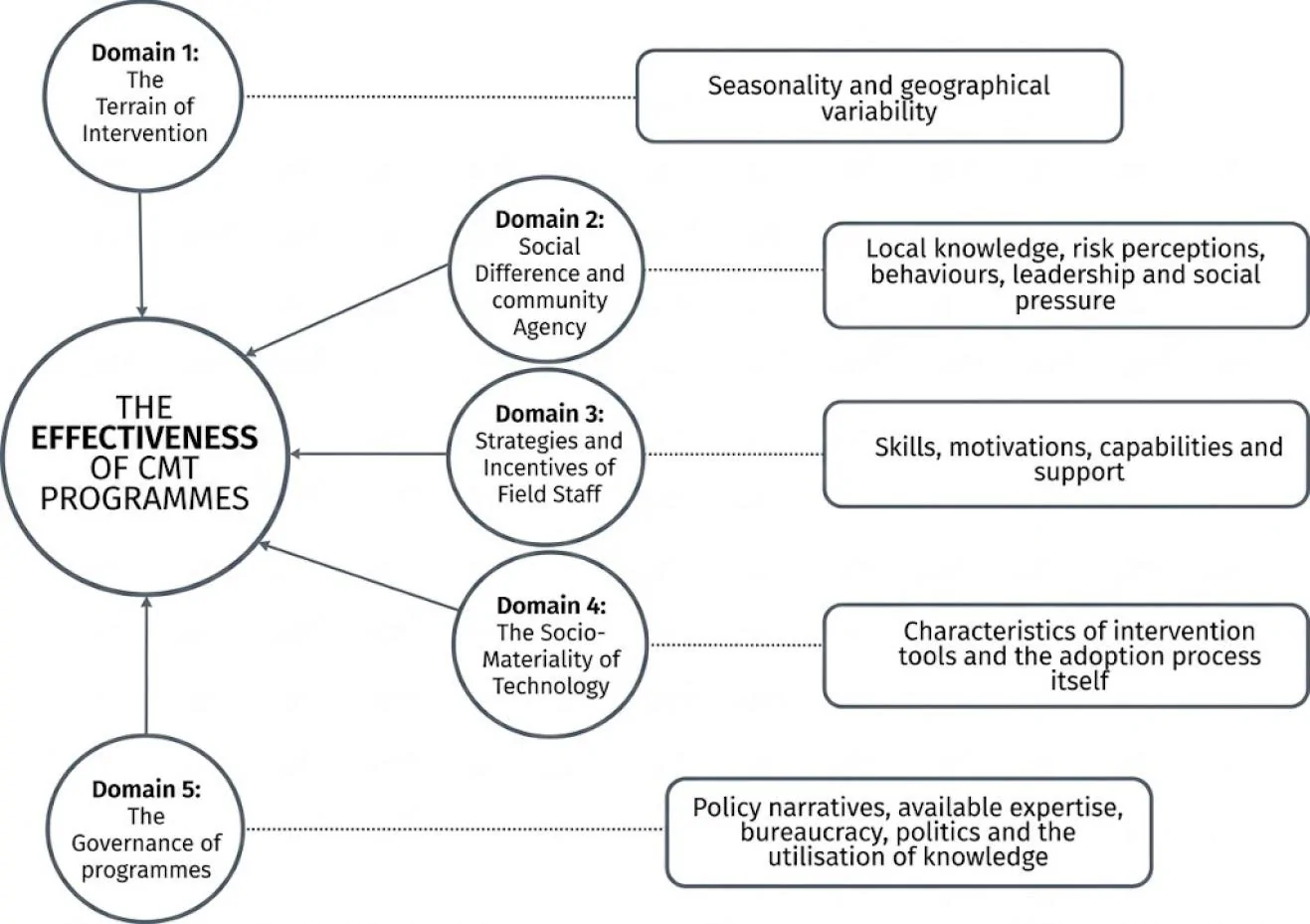
Analytical Framework (Adapted From Bardosh’s Framework of Global Health Delivery) for Assessing the Effectiveness of CMT Programmes in Ghana. Abbreviation: CMT, Chinese medical team.

 This framework has been applied to evaluate programme effectiveness in various global health settings.^31-35^ For example, Mtuy et al employed it to assess trachoma control efforts among pastoralist communities in Tanzania, revealing that socio-cultural mismatches between intervention design and community expectations undermined both uptake and sustainability.^31^

###  Researcher Positionality

 This study was conducted by a multidisciplinary team with diverse expertise spanning health policy, global health and clinical practice. Four scholars are from LMICs, three from Ghana and one from Sudan, with three affiliated with a Chinese medical university. Among them, one author is based at a Ghanaian teaching hospital currently hosting CMT programmes, while another holds dual affiliations with a Ghanaian teaching hospital and university, grounding their perspectives in local healthcare delivery and education. Complementing the local and regional perspectives are three Chinese scholars, whose research focuses on health policy and global health systems. This composition of the team brings together both academic and practitioner backgrounds, as well as local and international insights. Such diversity strengthens the study’s analytical depth and enhances its contextual relevance. It also enables a more balanced and reflexive interpretation of the contributions and complexities associated with CMT programmes in Ghana.

###  Study Sites, Participants, and Sampling

 Study sites were purposively selected to capture institutional diversity and contextual variation in the implementation of CMT programmes. Selection criteria included: (1) over 10 years of continuous CMT engagement; (2) representation of both urban and rural healthcare settings; and (3) national referral or specialised service functions. Based on these criteria, four institutions were included: Ghana’s Ministry of Health (MoH); Ledzokuku Krowor Municipal Assembly (LEKMA) Hospital, a 150-bed China-Ghana friendship facility in Accra; Korle-Bu Teaching Hospital (KBTH), a 2000-bed tertiary hospital in Accra; and Komfo Anokye Teaching Hospital (KATH), a 1200-bed tertiary hospital in Kumasi.^36-38^

 Prior to data collection, a stakeholder mapping^39^ was conducted by two authors to identify initial participants with direct experience in CMT-related activities since 2009. This process involved reviewing programme documents, such as Memorandums of Understanding (MoUs) and departmental records, to compile a list of potential informants. From this list, eleven individuals were purposively selected based on their institutional roles, depth of experiential knowledge and involvement in the planning, coordination or delivery of CMT services. To enhance the diversity of perspectives while maintaining relevance to the research objectives, a snowball sampling strategy was subsequently employed, allowing initial participants to recommend additional informants. The final sample comprised Ghanaian clinicians, hospital administrators, national-level policy-makers, Chinese medical staff and patients who had interacted with CMT services. Theoretical saturation was achieved after 18 interviews, as no new themes emerged in the final stages of data collection.^40^

###  Data Collection 

####  Informant Interviews

 A semi-structured interview guide developed in alignment with Bardosh’s framework was used to maintain consistency across interviews while allowing flexibility to probe emerging themes (Supplementary file 1, Table S2). To uphold methodological rigour, one author completed virtual training workshops on qualitative data collection in August 2021, followed by a refresher course in February 2024; these sessions covered face-to-face and digital interviewing techniques, with particular emphasis on mitigating bias in remote settings.

 Data collection occurred in two phases: The first phase was conducted between October and November 2021, consisting of 12 face-to-face interviews at KATH and KBTH. Phase two followed in February 2024 at the MoH and LEKMA, which involved 6 additional interviews, with 4 held virtually and 2 face-to-face. The virtual interviews were held via WhatsApp and Google Meet to reach geographically dispersed participants. All interviews took place in private settings, with each lasted between 30 and 75 minutes. With participant consent, sessions were audio-recorded; in instances where recording was declined, detailed notes were taken and later reviewed by participants to ensure accuracy.

 To minimise social desirability bias, and uphold ethical standards, the following measures were implemented prior to data collection: All participants received detailed information about the study and provided informed consent. They were assured of confidentiality, the voluntary nature of their participation, and their right to withdraw at any time without penalty. During the study, participants were reminded of their anonymity and encouraged to express their views openly to further reduce potential response bias. Following the interviews, all recordings were transcribed verbatim by an independent third party to avoid interpretive bias. Consistent with ethical research protocols, no financial incentives were offered to participants.

####  Document Review

 To triangulate and enrich the interview data, a structured review of policy and institutional documents was undertaken. Materials reviewed included MoUs, national health sector strategic plans, annual performance reports and administrative records related to CMT operations from 2009 to 2024. These documents were assessed using Scott’s four criteria for documentary analysis: authenticity, credibility, representativeness, and meaning.^41^ The review provided historical and institutional context for interpreting implementation processes, actor relationships and shifts in programme delivery over time.

###  Data Analysis

 Data were analysed using a directed content analysis approach,^42,43^ which integrates deductive categorisation based on predefined domains with inductive identification of emerging themes. The initial coding framework was structured around Bardosh’s model, specifically its five core domains, while remaining open to unanticipated patterns and insights. This hybrid strategy allowed the research team to capture both theory-informed and context-specific findings.

 Following established qualitative analysis methods,^44^ all transcripts were manually coded in Microsoft Excel. The coding matrix included columns for assigned domains, inductively derived themes and illustrative participant quotations. Coding was conducted collaboratively by three authors, who held regular discussions to address discrepancies and reach consensus on interpretive decisions.^45^ To enhance the rigour and validity of the findings, interview data were triangulated with documentary sources, including MoUs, annual performance reports and other institutional documents. Inconsistencies or gaps were addressed through weekly team consultations, which supported reflexive interpretation and contextual alignment.^44^

 Descriptive statistics were used to summarise participant demographics. All identifiers were anonymised using coded initials such as government official (GO) and medical professional-Ghana (MPG). This is in accordance with ethical research protocols.^40^ The final analysis produced a synthesis of thematic domains, supported by representative quotes and corroborating documentary evidence. Particular attention was given to insights from six participants whose narratives introduced novel dynamics not captured within the original framework. Confidentiality and data security were maintained throughout the research process.

## Results

###  Characteristics of Study Participants 

 A total of 18 participants were included in the final sample, following the withdrawal of two individuals due to unforeseen circumstances. The characteristics of this group are summarised in Table 1. The sample represented diverse stakeholder groups, including MoH representative (n = 1), institutional/department heads (n = 4), Ghanaian medical professionals (n = 7), Chinese medical professionals (n = 1), and patients (n = 5). Patient participants predominantly resided in rural or peri-urban regions, aligning with the demographic profile of populations reliant on the selected healthcare facilities.

**Table 1:** Characteristics of Study Participants (N = 18)

**Stakeholder Category**	**Number**	**Gender**	**Site**	**Years in Role/Position**	**Year of CMT Engagement**	**Interview Mode**
Government Official	GO001	Male	MoH	8 years	2021	Virtual
Institutional/Department Head	IDH001	Male	KATH	14 years	2014	In-person
	IDH002	Male	KATH	18 years	2014	In-person
	IDH003	Female	LEKMA	13 years	2014	Virtual
	IDH004	Male	KBTH	26 years	2014	In-person
Medical Professional (Ghanaian)	MPG001	Female	KATH	20 years	2014	In-person
	MPG002	Male	KATH	18 years	2014	In-person
	MPG003	Male	KATH	14 years	2014	In-person
	MPG004	Female	KATH	12 years	2014	In-person
	MPG005	Male	KATH	19 years	2014	In-person
	MPG006	Male	KATH	9 years	2014	In-person
	MPG007	Male	LEKMA	12 years	2014	Virtual
Medical Professional (Chinese)	MPC001	Female	LEKMA	11 years	N/A	Virtual
Patient (Ghanaian)	PG001	Female	KATH	N/A	2019	In-person
	PG002	Female	KATH	N/A	2014	In-person
	PG003	Male	KATH	N/A	2019	In-person
	PG004	Female	LEKMA	N/A	2024	In-person
	PG005	Female	LEKMA	N/A	2024	In-person

Abbreviations: CMT, Chinese medical team; GO, Government official; IDH, Institutional/Department Head; KATH, Komfo Anokye Teaching Hospital; KBTH, Korle-Bu Teaching Hospital; LEKMA, Ledzokuku Krowor Municipal Assembly; MoH, Ministry of Health; MPC, Medical professional (Chinese); MPG, Medical professional (Ghanaian); PG, Patient (Ghanaian); N/A, not available.

 In contrast, healthcare professionals, including CMT members, were primarily urban-based, reflecting the geographic distribution of Ghana’s tertiary care infrastructure. This participant composition enabled a multi-level exploration of CMT programme dynamics, integrating policy, frontline implementation and patient perspectives. Data triangulation across interviews (2009‒2024) and institutional documents traced the historical line of CMT programmes, revealing systemic challenges, operational adaptations and programmatic achievements for 15 years.

###  Thematic Domains

 The analysis identified seven thematic domains shaping the effectiveness of CMT programmes in Ghana. All five constructs from Bardosh’s framework (terrain of intervention, social difference and community agency, strategies and incentives of field staff, socio-materiality of technology and governance of interventions) emerged clearly through deductive analysis, confirming the framework’s applicability to this context. In addition, two inductively derived domains surfaced from participant narratives: patient-centred care, which captured expectations around respectful, responsive and culturally sensitive services; and sustainability in healthcare, which reflected concerns regarding long-term integration, system resilience and continuity of support. Together, these seven domains offer a comprehensive perspective on the operational and relational dynamics influencing CMT effectiveness in Ghana. A summary of these domains and their associated factors is presented in Table 2.

**Table 2:** Factors Shaping the Effectiveness of Chinese Medical Team Programmes in Ghana

**Domain**	**Factors**	**Strengths**	**Challenges**
Terrain of Intervention	Seasonal rotations and urban hospital placements Integration of CMTs into clinical workflows	Structured coordination High-priority cases scheduled Collaborative case management	Limited rural access Services unavailable outside CMT windows
Social difference and community agency	Cross-cultural collaboration through MoUs Peer influence in traditional Chinese medicine	Strong institutional partnerships High community trust in the efficacy of traditional Chinese medicine	Persistent communication barriers Occasional community mistrust due to language gaps
Strategies and incentives of field staff	Short-term CMT assignments Informal mentorship High intrinsic motivation of field staff	Local staff upskilled through clinical shadowing High patient satisfaction	Specialist shortages post-departure Lack of formal training pathways
Socio-materiality of technology	Introduction of advanced technologies Specialist-led procedures Inconsistent post-rotation maintenance	Complex procedures made locally available Improved clinical capability	Maintenance delays Supply shortages Dependence on Chinese technicians
Governance	Bilateral agreements and MoUs Strategic support from MoH Disparities in rural service provision	Facilitation of logistics and staff training Active policy support from key ministries	Policy-practice gaps in decentralisation Bureaucracy in import processes
Patient-centred care approach	Consistent care delivery Cultural sensitivity Clear communication via interpreters	Respectful and consistent care Increased patient confidence in treatment plans	Initial patient unfamiliarity Limited awareness among rural populations
Sustainability in healthcare	Progressive knowledge transfer Independent local practice Weak material sustainability post-departure	Retention of medical expertise Reduction in outbound medical tourism Enhanced hospital credibility	Inconsistent material support Financial constraints in sustaining introduced innovations

Abbreviations: CMT, Chinese medical team; MoH, Ministry of Health; MoU, Memorandum of Understanding.

####  The Terrain of Intervention

 Participants described a well-structured system for CMT integration into Ghanaian hospitals since 2009, supported by consistent stakeholder coordination, including pre-deployment briefings, orientation schedules and logistics planning. Hospitals prepared in advance for annual visits, aligning complex cases with the CMTs’ specialist skillsets. These preparations helped embed CMTs into everyday workflows:

 “*We assign a liaison officer…from preparing accommodation to scheduling orientation meetings before the Chinese teams arrive*” (IDH001).

 CMT specialists were perceived as effective collaborators within surgical and cardiology units, enabling hands-on skills transfer and co-management of patients. Local clinicians actively sought to learn from these teams:

 “*As a specialist in cardiology…, I work with the Chinese medical team in the management of patients locally*” (MPG001).

 However, programme design was shaped by seasonal and urban-centric deployments. Many participants, especially from peripheral regions, noted the limited availability of services outside Accra and Kumasi.

 “*Healthcare access is limited in some parts..., like the northern sector. Many people have to travel long distances to other locations…only when the Chinese teams are present” *(PG001).

 While structured, the model inadvertently disadvantaged rural populations and highlighted an urgent need for geographic redistribution and year-round availability.

####  Social Difference and Community Agency

 Strong institutional collaborations, anchored in MoUs and hospital-to-hospital partnerships, formed the backbone of programme legitimacy and sustainability. These agreements encouraged leadership commitment, staff buy-in and reciprocal learning opportunities:

 “*The Chief Executive...inked an MoU…send-off events and review meetings...showcase the institutional leadership’s dedication to the partnership”* (IDH001).

 CMTs introduced traditional Chinese medicine, which gained traction due to observed clinical outcomes and peer influence. Both clinicians and patients began embracing acupuncture and herbal techniques:

 “*The local medical staff learned…into the traditional Chinese medicine practice…helped them better deliver services to local patients”* (MPG002).

 “*After the acupuncture relieved my chronic pain, three neighbours followed me…now there’s always a queue”* (PG003).

 Yet communication barriers persisted despite translation efforts. Language mismatches led to misunderstandings, especially during patient interactions:

 “*Despite the Chinese team’s appreciation for local practices, the local population finds it challenging to understand Chinese communication” *(GO001).

 The social environment of learning and diffusion was strong, but misinterpretations and gaps in cultural mediation remained unresolved in some settings.

####  Strategies and Incentives of Field Staff

 The CMTs’ professionalism and patient-centred approach stood out. Patients often expressed emotional gratitude and reported high satisfaction levels:

 “*A score of 9/10…my operation was even enough for me”* (PG004).

 CMTs were also described as responsive to high patient loads and committed to co-working with Ghanaian clinicians. Local staff gained clinical exposure and informal training, particularly in surgical and diagnostic procedures:

 “*Their commitment to patient care and knowledge sharing is commendable… consistently reflects their professionalism and expertise”* (MPG006).

 This commitment was echoed by the CMT members themselves, who framed their work as a direct response to observed local need:

 “*We have been here almost half a year and see many Ghanaians who need healthcare but do not get it. So our medical team aims to do our best and take full advantage of our experience and expertise to offer quality service to the country…”* (MPC001).

 Nonetheless, short-term rotations and workforce imbalances remained a major constraint. After the CMTs’ departure, hospitals struggled to fill gaps, especially in emergency medicine and specialties like psychiatry and cardiothoracic surgery:

 “*Currently we lack trauma nurses… open heart surgeons and psychiatric care are lacking”* (IDH001).

 While the incentives and training were valuable, systemic staffing shortages limited sustainability.

####  Socio-materiality of Technology 

 Advanced equipment brought by CMTs played a transformative role in expanding the range of hospital procedures. Cardiac and minimally invasive surgeries became possible, creating access to life-saving interventions:

 “*Minimally invasive cardiac techniques…meant we could now perform complex repairs without open-heart surgery”* (MPG004).

 CMT visits were synchronised with case backlogs, allowing local teams to benefit from specialist inputs while avoiding costly overseas referrals:

 “*The Chinese team comes annually, we schedule all pending cases”* (IDH002).

 However, the inability to maintain or recalibrate specialised machines in the absence of Chinese technicians resulted in idle equipment and loss of functionality between missions:

 “*Specialised machine sat idle for months awaiting specialist calibration”* (MPG001).

 “*Once their supplies got finished, we struggled to sustain the new standards…had no choice but to revert to our old techniques” *(MPG003).

 These findings underscore the fragility of tech transfer without long-term maintenance plans or local spare part supply chains.

####  Patient-Centred Care Approach

 Patients consistently described CMTs as trustworthy, compassionate and attentive. Their reliability, especially in achieving surgery quotas and providing follow-up care, strengthened public confidence:

 “*They set out to operate 40 cases, and surprisingly, they did that”* (IDH004).

 Patients and clinicians alike noted that CMTs explained procedures clearly and treated patients with dignity. Respect for patient autonomy was frequently observed:

 “*They treated me with respect, explained everything, and most importantly, they delivered what they promised”* (PG002).

 “*I thought the doctors were too serious…but then I realised they just wanted to be very clear about my treatment”* (PG004).

 These culturally responsive interactions laid the foundation for long-term community trust and reduced hesitation among first-time users.

####  Sustainability in Healthcare 

 Long-term capacity development emerged as one of the programme’s strongest features. Participants reported a gradual transition from observational learning to independent practice:

 “*Initially, we observed, then assisted, and now perform certain procedures like cardiology independently”* (MPG001).

 Institutions reported reputational gains and a broader portfolio of clinical services following repeated CMT engagements:

 “*Expanded access to sub-specialist services, improved research capacity…our hospital’s profile as a tertiary facility has been enhanced” *(GO001).

 CMTs also curbed outbound medical tourism by enabling complex procedures locally:

 “*Their expertise…enabled treatment of complex cases… helped improve our hospital’s overall capacity”* (MPG005).

 Still, disruptions between rotations sometimes reversed progress. Staff reverted to old protocols due to lack of continuity or supply:

 “*When the Chinese teams depart…we sometimes revert to old practices”* (IDH003).

 Participants repeatedly called for more frequent deployments, particularly in remote areas:

 “*A desire for more regular visits of the CMT team… a more frequent presence is necessary” *(MPG002).

####  Governance 

 Governance of the CMT programme operated at multiple levels, from hospital logistics to bilateral policy dialogues. MoH officials and hospital leaders often streamlined permits and provided operational support to enable smooth deployment:

 “*Allocated additional funding, provided dedicated staff… ensured necessary logistics”* (IDH003).

 “*The Ministry of Health fast-tracked equipment import permits”* (GO001).

 Bilateral exchange agreements supported long-term training pipelines and built mutual institutional trust:

 “*The 2019 Memorandum…established a physician exchange programme where our specialists now receive annual training in China” *(IDH001).

 Still, implementation challenges were evident. Although national policies called for equitable access, CMT services remained clustered in a few urban hospitals:

 “*Policy states ‘nationwide coverage,’ but…majority of CMT activities occur in Accra and Kumasi”* (IDH004).

 Essential drug stockouts added further burden to patients, who described having to search outside facilities for prescriptions:

 “*Sometimes…it can be challenging to obtain the medicine on-site… I often go out of the facility and buy it myself”* (PG002).

 These governance challenges limited the programme’s equity and continuity goals despite strong intent.

####  Supplementary Insights From Documentary Evidence

 To complement interview findings and trace the institutional trajectory of CMT programmes in Ghana, the authors reviewed 15 years of programme-related documents, including annual reports, MoUs, newsletters and media releases. These sources were analysed to corroborate interview themes, such as governance arrangements, reported outcomes, and shifts in programme focus, and to provide historical context for stakeholder perceptions. Table 3 summarises key developments chronologically, highlighting how programme emphasis evolved from infrastructure and training in earlier years toward sustainability and local capacity in later periods.

**Table 3:** Key developments in Chinese Medical Team Programmes Based on Documentary Analysis (2009–2024)

**Year**	**Source Type**	**Institutions Involved**	**Reported Outcomes & Thematic Focus**
2009–2014	MoUs, internal reports, news media	• Ghana MoH • Chinese Embassy in Ghana • KBTH • Guangdong Provincial People’s Hospital	• Formalisation of bilateral health cooperation frameworks. • Initial deployment of surgical teams under “Heart to Heart” programme. • Introduction of advanced surgical equipment and short-term training workshops. • Emphasis on infrastructure and technology transfer.
2015–2019	Annual reports, newsletters, government publications	• KATH • LEKMA Hospital • CMT leader	• Expansion of specialist services (cardiology, orthopaedics, traditional Chinese medicine). • Increased outpatient and surgical outreach activities in urban centres. • Reported improvements in clinical mentoring and local staff upskilling. • Focus on clinical integration and service scale-up.
2020–2024	Annual reports, policy briefs, institutional websites, media releases	• All study sites (MoH, KATH, KBTH, LEKMA)	• Adaptation to COVID-19, telemedicine consultations, personal protective equipment donations, and vaccine diplomacy. • Growing emphasis on sustainability, local capacity development, and retention of skills. • Documented reduction in outbound medical tourism for certain specialties. • Increased calls for rural outreach and longer-term rotations.

Abbreviations: CMT, Chinese medical team; KATH, Komfo Anokye Teaching Hospital; KBTH, Korle-Bu Teaching Hospital; LEKMA, Ghana-China Friendship Hospital; MoH, Ministry of Health; MoU, Memorandum of Understanding.

## Discussion

###  Factors Influencing the Effectiveness of CMT Programmes

 This study examined the effectiveness of CMT programmes in Ghana through a socio-anthropological lens, guided by Bardosh’s framework for global health delivery,^30^ revealing a multidimensional interplay of clinical service delivery, institutional arrangements and sociocultural dynamics. To contextualise these findings, the study considers the health system landscape into which CMTs are deployed. Ghana’s health system, while demonstrating resilience, contends with well-documented structural challenges: a maldistribution of the health workforce favouring urban centres, persistent resource constraints, a double burden of communicable and non-communicable diseases, and gaps in specialist care, particularly in surgical disciplines and rural areas.^22-25^ Within this context, and aligned with Ghana’s hospital governance structure, the positioning of CMTs primarily in tertiary urban hospitals both enables technical efficiency and constrains equity-oriented service delivery.

 In line with the adapted framework, the analysis confirms that CMTs in Ghana demonstrate both strategic relevance and operational complexity. Their presence helps address key health system gaps, notably in specialised surgical care such as cardiothoracic and minimally invasive procedures, short-term human resource reinforcement, technology transfer and areas where local capacity is limited; findings that echo previous research in sub-Sahara Africa and beyond.^3,10,46^ However, unlike traditional assessments that focus narrowly on medical outputs, this study foregrounds relational factors such as patient trust, cross-cultural communication and staff collaboration as central to the success of these transnational programmes.

 Importantly, the analysis adds two inductively derived domains, patient-centred care and sustainability in healthcare, to the existing framework. These reflect concerns over continuity, local capacity and the long-term viability of externally initiated interventions. While patients widely perceived CMTs as competent and compassionate, limitations in linguistic mediation and short team rotations hindered service continuity and institutional integration. Similar critiques have been raised in other LMIC contexts where temporary medical aid missions struggle to embed into local systems.^47-49^ Additionally, vertical interventions further highlight the risks of such fragmented approaches, which often prioritise short-term outputs over systemic integration.^50^

###  Implications

 At the governance level, findings illustrate both opportunities and fragilities. Although bilateral agreements and MoUs have fostered structured collaboration, their translation into equitable service distribution, particularly for underserved regions, remains uneven. The reliance on centralised urban hospitals for CMT operations may inadvertently deepen urban-rural disparities, despite policy intentions of nationwide coverage. To address Ghana’s top health priorities, such as reducing mortality, combating infectious diseases, and managing the growing burden of non-communicable diseases, CMT integration must be strategically aligned. This requires moving beyond ad-hoc placements to a needs-based deployment model.

 From a sustainability perspective, the CMT model demonstrates both promise and risk. On one hand, it supports capacity building, knowledge exchange and reduced patient outmigration for surgical care. On the other hand, these benefits are often time-bound and dependent on consistent team rotation, equipment maintenance and supportive infrastructure. Without robust local investment and systemic integration, there is a risk of stagnation or dependency once CMTs withdraw. These dynamics align with wider critiques of vertical health interventions that lack embeddedness within host systems.^51,52^

 Furthermore, the findings align with sustainability science by illustrating the importance of institutional co-evolution and resilience in health partnerships.^53,54^ Sustainable health interventions must go beyond temporary fixes to support adaptive learning, mutual accountability and resource continuity.^55^ This study advocates a shift from transactional aid to co-constructed partnerships, where relational sustainability is more important than mere outputs. It involves treating CMTs as learning ecosystems, where mutual adaptation, not just technical fixes, builds long-term resilience.

###  Strengths and Limitations

 A key strength of this study lies in the diverse, multidisciplinary composition of the research team, which included scholars and practitioners from both Ghana and China. This diversity enabled balanced analysis and contextual sensitivity, particularly in interpreting cross-cultural dynamics and stakeholder experiences. Additionally, the study employed methodological triangulation by combining in-depth interviews with a structured review of institutional documents, enhancing the validity and historical depth of the findings.

 Despite these strengths, several limitations should be noted. Social desirability bias may have influenced participant responses, especially given the diplomatic nature of the CMT programme.^56^ The limited geographic coverage focused primarily on two urban centres, which may constrain generalisability to more remote or under-resourced settings. Language barriers also affected the clarity of some patient narratives, even with interpreter assistance.

## Conclusions

 CMTs in Ghana offer tangible contributions to clinical service delivery and bilateral health cooperation. Yet, their effectiveness hinges not only on technical delivery but on trust-building, continuity and local system engagement. By adapting Bardosh’s framework and integrating patient-centred and sustainability dimensions, this study contributes a more nuanced lens for evaluating medical diplomacy. Strengthening these collaborations will require moving beyond short-termism toward shared ownership and institutional embedding, ensuring that external partnerships genuinely contribute to resilient and equitable health systems.

## Acknowledgements

 The authors sincerely thank all individuals who contributed to this study, including health workers and GOs, for their time, insights, and willingness to share their experiences. They also acknowledge the support of Southern Medical University and the Chinese Government Doctoral Scholarship for supporting the doctoral studies of the first author.

## Disclosure of artificial intelligence (AI) use

 AI tools were used only for language editing and polishing. All scientific content, analysis, and interpretations were developed by the authors.

## Ethical issues

 Ethical approval for this study was initially obtained from the Institutional Review Board of Southern Medical University, China (Approval No.: N.Y.LS. (2021) No. 014). In Ghana, ethical clearance was also secured from KATH, KBTH, and LEKMA Hospital under the Ghana Health Service, a government agency under the MoH responsible for administering public health services and implementing national health policies. Prior to data collection, all participants were provided with detailed information about the study and gave written informed consent. They were assured of the confidentiality of their responses, the voluntary nature of their participation and their right to withdraw from the study at any time without penalty or consequence.

## Conflicts of interest

 Authors declare that they have no conflicts of interest.

## Disclaimer

 The authors confirm that this work is original and has not been published elsewhere, nor it is currently under consideration for publication elsewhere.

## Supplementary files


Supplementary file 1 contains Tables S1-S2.
