# Supplementary material for: Understanding the Factors Shaping the Effectiveness of Chinese Medical Team Programmes in Ghana: A Qualitative Study Using Bardosh’s Framework of Global Health Delivery
Source: Int J Health Policy Manag. 2026 May 18;15:9320. doi: 10.34172/ijhpm.9320 (PMC13498950; doi:10.34172/ijhpm.9320)
Supplement: Supplementary file 1 [file ijhpm-15-9320-s001.pdf]

Article title: Understanding the Factors Shaping the Effectiveness of Chinese Medical Team Programmes in Ghana: A Qualitative Study Using Bardosh's Framework of Global Health Delivery

Journal name: International Journal of Health Policy and Management (IJHPM)

Authors' information: Emmanuel Kwasi Afriyie[1,2,3], Samuel Egyakwa Ankomah[3,4], Munawar Harun Koray[1,2,5], Sara Sulieman[1,2,6], Huijuan Liang[7], Dadong Wu[8]*, Dong (Roman) Xu[1,2,9]*

[1]School of Public Health, Southern Medical University, Guangzhou, China.

[2]Acacia Lab for Implementation Science, School of Health Management, Southern Medical University, Guangzhou, China.

[3]Komfo Anokye Teaching Hospital, Kumasi, Ghana.

[4]Department of Management, University of Cape Coast, Cape Coast, Ghana.

[5]Upper West Region Health Directorate, Wa, Ghana.

[6]Department of Pharmacy Practice, Faculty of Pharmacy, University of Khartoum, Khartoum, Sudan.

[7]School of Healthcare Management, Inner Mongolia Medical University, Hohhot, China.

[8]Shenzhen Maternity and Child Healthcare Hospital, Southern Medical University, Shenzhen, China.

[9]SMU Institute for Global Health (SIGHT) and Center for World Health Organization Studies, School of Health Management and Dermatology Hospital of Southern Medical University (SMU), Guangzhou, China.

***Correspondence to:** Dadong Wu; Email: dadongwu@smu.edu.cn & Dong (Roman) Xu; Email: romanxu@i.smu.edu.cn

Citation: Afriyie EK, Ankomah SE, Koray MH, et al. Understanding the factors shaping the ffectiveness of Chinese medical team programmes in Ghana: a qualitative study using Bardosh's framework of global health delivery. Int J Health Policy Manag. 2026;15:9320. doi:10.34172/ijhpm.9320

Supplementary file 1

Table S1**:** Standards for Reporting Qualitative Research (SRQR) Checklist

Standards for Reporting Qualitative Research (SRQR)		
No.	Topic	Page (section) in manuscript
	**Title and abstract**	
S1	Title	P1
S2	Abstract	P1
	**Introduction**	
S3	Problem formulation	P2

S4	Purpose or research question	P2
	**Methods**	
S5	Qualitative approach and research paradigm	P2
S6	Researcher characteristics and reflexivity	P2-3
S7	Context	P3
S8	Sampling strategy	P3
S9	Ethical issues pertaining to human subjects	P4 and P9
S10	Data collection methods	P3-4 and Supplementary file 1, Table S2
S11	Data collection instruments and technologies	P3-4
S12	Units of study	P4
S13	Data processing	P4
S14	Data analysis	P4
S15	Techniques to enhance trustworthiness	P4
	**Results/findings**	
S16	Synthesis and interpretation	P4-7 and Tables 2-3
S17	Links to empirical data	P4-7
	**Discussion**	
S18	Integration with prior work, implications, transferability, and contribution(s) to the field	P7-8
S19	Limitations	P8
	**Other**	
S20	Conflicts of interest	P9
S21	Funding	P9

Table S2**:** Key informant interview guide, informed by Bardosh's framework of global health delivery

Domains	Constructs	Interview Questions
**Terrain of Intervention**	Seasonality and geographical variability	Q1: What is your role in the team? What elements and parts of your work require collaboration or communication with the CMT? Please describe what you do daily, especially the parts that involve collaboration with the CMT.
		Q2: How well do you think the local medical team and the CMT work together? Describe the relationship between the local medical staff, leadership (head of the local department/medical facility) and the CMT, giving one or two examples.
		Q3: What contributions has the CMT made to local health care? Please give 1 or 2 examples of this or tell 1 or 2 impressive experiences, events, people, etc., during your service.
		Q4: In what areas do you think the CMT contribution/service effectiveness is still insufficient? What would you suggest to improve this? Please give 1 or 2 examples of this or tell 1 or 2 impressive experiences, events, people, etc.
		Q5: Do you know what activities/services the CMT provides to your region/country?
**Social Difference and Community Agency**	Local knowledge, risk perceptions, behaviours, leadership and social pressure	Q1: What do you think about the CMT? Or what is your overall impression of the CMT?
		Q2: What do you think are the main difficulties and challenges that local people will encounter in receiving services from CMT? Please enumerate or give examples.
		Q3: Which of the above difficulties and challenges are yet to be addressed by the CMT? What do you think can be done to improve or solve them?
		Q4: Thinking about the local residents/patients, what would be their primary health needs?
		Q5: To what extent do you think the CMT understands the needs of local health services? (On a scale of 1-10, with 1 being the lowest and 10 being the highest)? Why or why not?
		Q6: Do you think the local residents would prefer to seek services provided by local medical staff or the CMT? Why or why not?

		Q7: How well do the local cultural values and social norms fit with those of China? Which aspects of cultural practices do you think are the most different? How does it affect the effectiveness of CMT implementation?
**Strategies and Incentives of Field Staff**	Skills, motivations, capabilities and support	Q1: Would you recommend the CMT services to local residents? Why or why not?<br><br>Q2: What type of health personnel do you think Chinese medical aid teams sending abroad would be more helpful to you?<br><br>Q3: How would you characterise the level of support from the leader of local institutions for your team's involvement in the CMT projects? “Probe.” Can you give some specific examples of support (e.g., extra staff, devoted time from existing staff, extra space, more funding, etc.)?<br><br>Q4: What role do you think the CMT has played, or what impact has it had?<br><br>Q5: What support and inspiration have you and your colleagues received from your country, city, workplace, or China as a result of your participation in the CMT collaboration?<br><br>Q6. Do you think these are effective in motivating health personnel to participate in foreign CMT? Why or why not?<br><br>Q7. What do you think is feasible, or what needs to be changed to improve the incentives for foreign CMT?
**Socio-materiality of Technology**	Characteristics of intervention tools and the adoption process itself	Q1: What do you think needs to be done in the future to improve the health of the local population, or what are some measures that would enable better collaboration between local health teams and the CMT?
**Governance**	Policy narratives, available expertise, bureaucracy, politics and the utilisation of knowledge	Q1: Are there any policies, regulations or guidelines in the country that would affect the continued implementation of the CMT locally?<br><br>Q2: Do you know about medical aid teams from other countries in your area? What do you think about the service of medical aid teams from other countries? (Skip the question if you do not know.)<br><br>Q3: What efforts and changes do you think the government and regulators can make in response to the suggestions you mentioned above?
